# Autonomy, quality of care, life satisfaction, and depressive symptoms among long-term care residents: a comparative study of selected facilities in Poland and Slovakia

**DOI:** 10.3389/fpubh.2026.2015119

**Published:** 2026-10-30

**Authors:** Bożena Majchrowicz, Katarzyna Tomaszewska, Ĺudmila Matulníková, Helena Kadučáková, Krystyna Kowalczuk

**Affiliations:** 1 Faculty of Health Sciences and Psychology, Institute of Nursing, Collegium Medicum, University of Rzeszów, Rzeszów, Poland; 2 Faculty of Health Care, Bronisław Markiewicz State Academy of Applied Sciences, Jarosław, Poland; 3 St. Ladislaus Faculty of Health and Social Work in Nové Zámky, St. Elizabeth University of Health and Social Work, Bratislava, Slovakia; 4 Faculty of Health Sciences, Department of Nursing, Catholic University in Ružomberok, Ružomberok, Slovakia; 5 Department of Health Care Sciences, Faculty of Humanities, Tomas Bata University in Zlin, Zlín, Czechia; 6 Department of Integrated Medical Care, Medical University of Białystok, Białystok, Poland

**Keywords:** autonomy, older adults, life satisfaction, long-term care, quality of care

## Abstract

**Introduction:**

The quality of long-term care encompasses not only safety and meeting the health needs of residents, but also the opportunity to preserve autonomy, agency, and influence over daily life. The aim of the study was to evaluate the association between selected aspects of institutional care quality, autonomy, and functioning of older adults with life satisfaction and the severity of depressive symptoms, taking into account differences between residents of facilities in Poland and Slovakia.

**Material and methods:**

A multicenter, cross-sectional comparative study was conducted involving 322 residents of six long-term care facilities: 127 in Poland and 195 in Slovakia. A original study questionnaire assessing selected aspects of care quality and autonomy was used alongside standardized scales: ADL, IADL, SWLS, and GDS-15. The analysis utilized the Mann–Whitney *U*-test, Pearson's chi-squared test, and Spearman's rank correlation. Additionally, multivariable analyses were performed using a General Linear Model (GLM/UNIANOVA), incorporating study group, sex, length of stay in the facility, and the circumstances of the admission decision.

**Results:**

Among the residents of the six participating facilities, individuals residing in Slovakia rated most of the analyzed aspects of care higher, including living conditions, safety, staff availability, social contacts, and engagement activities. The greatest differences concerned autonomy, particularly the influence on daily routine organization and participation in decisions regarding their own care (both *p* < 0.001). In the study sample, residents of the Slovakian facilities also obtained higher life satisfaction scores (SWLS: 23.33 vs. 19.98; *p* < 0.001) and lower scores for depressive symptom severity (GDS-15: 5.50 vs. 7.30; *p* < 0.001) compared to residents of the Polish facilities.

**Conclusions:**

The observed differences in the assessments of care quality, autonomy, and psychosocial wellbeing pertain to the residents of the participating facilities and cannot be generalized to the national long-term care systems in Poland and Slovakia. The results highlight the need for further research into the significance of autonomy, shared decision-making, and social relationships for the wellbeing of residents in institutional care.

## 1 Introduction

The progressive aging of the population and the increase in the number of individuals requiring ongoing support in daily functioning represent some of the most critical challenges facing contemporary healthcare systems and social policy. As life expectancy increases, the prevalence of multimorbidity, functional limitations, and the demand for long-term assistance in performing basic and complex activities of daily living also grow. A consequence of these processes is the rising importance of long-term care (LTC), provided both in home settings and in 24-h residential care facilities. Modern approaches to LTC extend beyond ensuring safety and meeting basic health needs. Increasing attention is paid to residents' quality of life, respecting their preferences, allowing personal choice, and preserving agency despite growing dependence on assistance from others (1, 2).

A long-term care facility is not merely a venue for healthcare and caregiving services; it becomes the resident's living environment. For this reason, quality of care should be viewed more broadly than simply through the lens of service availability or procedural correctness. Living conditions, privacy, feelings of safety, staff availability, responsiveness to residents' needs, communication clarity, respect demonstrated by staff, and opportunities to maintain social relationships and engage in activities are equally vital (2–4). Literature reviews indicate that both individual resident characteristics and modifiable facility environment features can be associated with older adults' quality of life. Particular importance is attributed to an activity-conducive environment, social support, activity availability, and care organization that enables the fulfillment of individual needs (3, 4).

In this context, a key component of person-centered care is resident autonomy. Admission to a care facility often requires adapting one's existing lifestyle to the institutional rhythm, which can constrain decision-making regarding daily activities. However, autonomy does not strictly imply complete functional independence. Even an individual requiring assistance can retain the ability to express preferences, make decisions, and influence the manner and timing of support provided. In practice, autonomy can manifest as the ability to decide on daily scheduling, select activities, participate in care-related decisions, and have personal habits and needs respected (5, 6).

Research on person-centered care indicates that supporting autonomy requires an appropriate organizational culture and positive relationships between staff and residents. Van Loon et al. emphasized the significance of shared decision-making, appropriate staff-resident relationships, organizational systems that support the resident, and creating an environment that enables true participation of the older adult in shaping their life (5). Simultaneously, observations of daily interactions within facilities suggest that opportunities to support autonomy are not always fully utilized, particularly for residents with higher dependency levels or cognitive impairments (6). A recent review of person-centered care models confirms that autonomy and dignity, individualized care, social engagement, a home-like environment, and family involvement are fundamental elements of models that foster improved experiences for LTC residents (7).

An essential aspect of assessing older adults' functioning remains life satisfaction, which constitutes the cognitive component of subjective wellbeing. It reflects an individual's overall evaluation of their life relative to their expectations, needs, and adopted standards. For residents of long-term care facilities, this evaluation may relate not only to health status and functional capacity, but also to the character of the institutional environment and the ability to maintain control over their life. Studies among institutionalized older adults highlight the importance of functional independence for quality of life, alongside links between life satisfaction, individual psychological resources, and health behaviors (8, 9). In a study comparing facility residents in Poland and Germany, quality of life remained closely tied to the participants' level of independence (8).

Equally significant are depressive symptoms among LTC residents. Institutionalization can coincide with the loss of former social roles, reduced contact with previous social circles, declining physical capacity, multimorbidity, and increased reliance on others. All these factors can heighten vulnerability to psychological decline. Longitudinal studies demonstrate that depressive symptoms can persist long after admission, with varying trajectories among residents (10). Recent data from Polish residential care facilities also show a high prevalence of depressive symptoms and their association with limited family contact, multimorbidity, polypharmacy, and lower functional independence (11).

The impact of depression in the LTC population is further evidenced by numerous studies evaluating interventions aimed at symptom reduction. Systematic reviews point to potential benefits from psychosocial interventions, physical activity, reminiscence therapy, and other non-pharmacological methods (12–15). For instance, a meta-analysis on resistance band exercises showed simultaneous positive effects on physical functioning, sleep quality, and depressive symptoms among LTC residents (13). Reminiscence therapy has also been shown to help reduce depressive symptoms and improve select quality of life aspects (14). These findings indicate that a resident's mental health remains linked to physical and social functioning as well as the nature of daily facility experiences.

The quality of resident-staff relationships appears particularly vital. Research demonstrates that nurse-patient interactions are an essential component of residents' quality of life, while loneliness, anxiety, and depressive symptoms can negatively impact it (16). Concurrently, residents experiencing depressive symptoms express diverse needs; their ability to communicate these needs depends on staff availability, patience, willingness to listen, and treating the resident as a partner. Unmet needs and communication barriers can thus form a critical part of the institutional care experience (17). Qualitative studies also show that care perceived as lacking respect can induce feelings of insignificance, sadness, and suffering in older adults, reinforcing the crucial role of dignity and relational care quality (18).

Evaluating the relationships between care quality, autonomy, and psychological functioning is especially relevant in international comparative research. European long-term care systems vary in organization, funding, service availability, and integration between health and social sectors. Current European analyses point to differences regarding care options and the degree to which person-centered care principles are realized (1). Poland and Slovakia share partially similar historical, social, and cultural contexts, yet LTC system organization and development operate under distinct institutional frameworks. In Slovakia's case, studies have pointed to fragmented governance and financing, as well as insufficient coordination between healthcare and social services (19, 20).

Direct comparative studies of older adult populations in Poland and Slovakia remain scarce. Kowalczyk et al. (21), surveying 1,534 community-dwelling adults aged ≥60 in Poland and Slovakia, observed cross-country differences in overall quality of life, health satisfaction, and physical, psychological, and environmental dimensions. However, that study focused on community-dwelling seniors rather than individuals in 24-h institutional care.

Despite growing literature on quality of life, depression, and person-centered care, significant knowledge gaps persist. Much published research focuses on single outcomes—quality of life, depressive symptoms, functional independence, or specific interventions—whereas the relationships between subjectively rated daily care quality, resident autonomy, life satisfaction, and depressive symptoms are less frequently analyzed within a unified framework (3, 7, 12). Available data on autonomy often derive from qualitative research, observational studies, or Western European healthcare settings (5, 6). Relatively little remains known about whether the ability to influence daily routines and participate in care decisions associates with life satisfaction and depressive symptom severity among institutionalized residents in Central European countries.

Notwithstanding existing studies on Polish institutionalized residents (8, 9, 11) and comparative studies of community-dwelling seniors in Poland and Slovakia (20), direct comparative research integrating the resident's perspective on care quality and autonomy with life satisfaction and depressive symptom assessments in LTC facilities is lacking. Comparing these two populations can provide insights not only into variable level differences, but also into whether relationships between care experience, autonomy, and psychosocial functioning exhibit similar patterns across both countries.

Accordingly, the aim of this study was to evaluate the association between selected aspects of institutional care quality, autonomy, and older adult functioning with life satisfaction and depressive symptom severity, accounting for differences between long-term care facility residents in Poland and Slovakia.

## 2 Material and methods

### 2.1 Research design

The study was a multicenter, cross-sectional, comparative observational study conducted using a diagnostic survey method. The study included residents of six purposively selected long-term care facilities, three each in Poland and Slovakia. The aim was to compare selected characteristics of the residents in the participating facilities and to assess the relationships between the subjective evaluation of care quality, autonomy, functional capacity, life satisfaction, and the severity of depressive symptoms. The adopted study design allowed for the comparison of the two resident groups, but did not provide a basis for drawing representative conclusions regarding the national long-term care systems.

### 2.2 Research tools

The study utilized an original survey questionnaire and four standardized instruments evaluating functional capacity, life satisfaction, and depressive symptoms: Katz Index of Independence in activities of daily living (ADL), Lawton instrumental activities of daily living scale (IADL), Satisfaction With Life Scale (SWLS), and the 15-item Geriatric Depression Scale (GDS-15). The selected toolset aligned with the study framework.

#### 2.2.1 Author's survey questionnaire

An custom questionnaire was developed specifically for this study to obtain subjective evaluations of selected aspects of care quality, living conditions, and autonomy among long-term care residents. Prior to data collection, formal content validity assessment by an expert panel and pilot testing were not conducted. Consequently, individual items were analyzed as discrete indicators of the assessed care dimensions rather than as components of a validated psychometric scale.

The questionnaire was developed specifically for this study. The first part contained items regarding gender, length of stay in the current facility, decision-making circumstances surrounding admission, and frequency of contact with relatives. The second part assessed subjective evaluations of selected care and stay aspects, spanning: living conditions/environment, care organization/staffing, autonomy/shared decision-making, social contacts/activation, stay-related burden/experiences, and overall care rating. Items were generally rated on a 5-point scale, where higher scores indicated a more favorable assessment (except for pain frequency, where higher scores represented greater frequency).

Autonomy and shared decision-making were measured via two questions regarding influence over one's daily routine (e.g., meal and rest times) and participation in care decisions. Social contact and activation covered family contact frequency, available activity offerings, and self-reported activity participation. Additional items recorded pain frequency, sleep quality, falls in the preceding 6 months, overall care satisfaction, and willingness to recommend the facility. An open-ended question invited participants to list care aspects most requiring improvement.

To ensure cross-national consistency, the original questionnaire underwent linguistic and cultural adaptation. The Slovak version was created following a forward-backward translation procedure. Conceptual and semantic discrepancies were resolved by a joint Polish-Slovak research team through consensus, establishing semantic and cultural equivalence.

#### 2.2.2 Katz index of independence in activities of daily living (ADL)

Basic functional independence was evaluated using the Katz ADL scale (22). The Slovak group used the Slovak language ADL version established in domestic gerontological literature (23), while the Polish cohort was assessed using the ADL scale embedded within Comprehensive Geriatric Assessment (CGA) protocol.

The scale assesses six basic tasks: bathing, dressing, toileting, transferring, continence, and feeding. Independent execution yields 1 point; dependence yields 0 points (total range: 0–6). Scores of 5–6 denote preserved independence, 3–4 moderate impairment, and ≤ 2 severe impairment. Identical scoring and interpretation were applied across both country cohorts to enable direct comparison.

The total score of the ADL scale ranged from 0 to 6 points, with higher values indicating greater independence in performing basic activities of daily living. In the statistical analyses, only the total ADL score was used and treated as a quantitative variable, without dividing the participants into categories of functional capacity. An identical scoring method was applied in both study groups, allowing for a direct comparison of the obtained results.

#### 2.2.3 Lawton instrumental activities of daily living scale (IADL)

Complex activities were assessed using the Lawton IADL scale (24). Items were scored dichotomously (1 = independent, 0 = dependent), with higher totals indicating greater functional independence. The Slovak component used the “Lawton–Brodieho test IADL” standardized for Slovak comprehensive geriatric assessments (25).

The instrument covers eight complex tasks: telephone use, shopping, food preparation, housekeeping, laundry, mode of transportation, medication management, and handling finances. Scoring criteria were standardized across both groups.

The interpretation of instrumental activities of daily living (IADL) scores accounted for the specific context of institutional care. Certain complex daily activities, such as meal preparation, shopping, or housekeeping, may not be performed independently by residents due to the operational organization of the facility. Consequently, non-performance of a given activity does not necessarily indicate an inability to execute it. Therefore, IADL scores were interpreted with caution, considering the potential confounding effect of the institutional environment on the assessment of functional independence.

#### 2.2.4 Satisfaction with life scale (SWLS)

Life satisfaction was assessed using the Satisfaction With Life Scale (SWLS), developed by Diener, Emmons, Larsen, and Griffin (26). The tool consists of five statements referring to a global cognitive evaluation of one's own life. Each statement is rated on a 7-point Likert scale, ranging from 1 – “strongly disagree” to 7 – “strongly agree.” The total score ranges from 5 to 35 points, with a higher score indicating greater life satisfaction. This response format was used in the study.

In the Polish portion, the Polish adaptation of the SWLS developed by Juczyński was applied. In the normalization study involving 371 individuals, the Polish version demonstrated satisfactory internal consistency (Cronbach's α = 0.81), and the test–retest stability coefficient after six weeks was 0.86 (27). These data are also confirmed by the Psychological Test Laboratory of the Polish Psychological Association.

In the Slovak portion, the Slovak language version of the SWLS was used. The translation of the scale into Slovak was developed by Halama and Dědová (28). Studies using this adaptation confirmed its good internal consistency; a recent Slovak sample yielded a Cronbach's α of 0.89.

#### 2.2.5 Geriatric depression scale (GDS-15)

The severity of depressive symptoms was assessed using the 15-item Geriatric Depression Scale (GDS-15), a shortened version of the tool developed for screening depressive symptoms in older adults (29, 30). In the study conducted in Slovakia, the Slovak language version of the GDS-15 was used (31), whereas in Poland, the Polish version of the scale employed within the Comprehensive Geriatric Assessment (CGA) was applied.

The GDS-15 consists of 15 questions requiring a “yes” or “no” response. Each response indicating the presence of depressive symptoms is scored as 1 point, while a response indicating no presence of symptoms receives 0 points. The total score ranges from 0 to 15 points, with higher values indicating greater severity of depressive symptoms. It should also be emphasized that the GDS-15 is a screening tool and does not provide a basis for establishing a clinical diagnosis of depression. In both study groups, an identical scoring and interpretation method was applied, enabling direct comparison of the results. In statistical analyses, only the total scale score was used. Cut-off points were not applied, nor were participants categorized by the severity of depressive symptoms.

### 2.3 Participants

Participating facilities were selected purposively, taking into account feasibility and facility administration approval. The research took place in three long-term care facilities in Poland and three senior homes in Slovakia. In each facility, residents meeting eligibility criteria were invited to participate. Participation was voluntary; the final sample comprised residents who provided informed consent and completed the research tools correctly. The study was conducted in six long-term care facilities, three in each country. For the purpose of statistical analysis, participant data were aggregated into two national groups (PL and SK), without retaining individual facility identifiers within the analytical dataset. Consequently, the individual resident served as the unit of analysis, and comparisons were performed at the national group level.

No formal preliminary sample size calculation was performed prior to the study. Sample size depended on available residents across the six participating facilities who met eligibility criteria and consented during the study period. 200 questionnaires were distributed to each national study arm, with final data gathered from 322 individuals (127 residents in Poland, 195 in Slovakia). With these sample sizes, a two-tailed significance level of α = 0.05 and 80% power allows for the detection of an intergroup effect size of approximately *d* = 0.32 (small to moderate). Data collection in Poland occurred from May to late June 2026, while the Slovak component took place in June 2026.

Residents of long-term care facilities were eligible for the study if they met the established inclusion criteria, which comprised being aged 65 years or older, having the capacity to understand the questionnaire items, and providing informed consent to participate. Specific ages or age categories were not recorded during data collection, precluding the reporting of a detailed age breakdown for the study groups.

Exclusion criteria included the absence of informed consent, a health status that precluded participation, and severe cognitive impairment that prevented comprehension of the questions or meaningful participation. Participants retained the right to skip any question without providing a reason. Non-response was not grounds for exclusion from the overall study; rather, incomplete entries resulted in exclusion solely from analyses requiring complete scores for the respective scale.

### 2.4 Data collection procedure

The study was conducted using paper-based questionnaires. Participation in the study was voluntary and anonymous. Prior to the study, potential participants were informed about its purpose, implementation rules, confidentiality of the data obtained, and the option to withdraw from participation at any stage without providing a reason and without affecting the scope or quality of care received. Following informed consent, the study was conducted using a prepared set of tools. The same study design and a corresponding scope of variables were applied in both the Polish and Slovak parts, enabling a comparative analysis of the two groups.

After obtaining approval from the management of the facilities in each country, 200 questionnaire copies were distributed per country, with a request to make them available to residents meeting the inclusion criteria. Properly completed questionnaires were obtained from 322 residents of long-term care facilities, including 127 individuals residing in facilities in Poland and 195 individuals residing in facilities in Slovakia. This represents an overall response rate of 80.5%, with 63.5% in Poland and 97.5% in Slovakia.

### 2.5 Ethical procedure

The study was conducted in accordance with the principles of the Declaration of Helsinki and ethical guidelines for conducting research involving human participants. Participation in the study was voluntary and anonymous. Prior to entering the study, participants were informed of its purpose and procedure, confidentiality rules, and the right to withdraw from participation at any stage without providing a reason. All participants gave informed consent to participate in the study. In the Polish part, the implementation of the study was approved by the Bioethics Committee of PANS in Przemyśl, approval no. 2/2024. The Slovakian part of the study was conducted in three facilities providing social and care services for older adults (*zariadenia pre seniorov*), rather than in medical facilities. The study was observational and non-interventional, and data were collected using questionnaires. No medical procedures were performed, and there was no interference with the provision of services or care. Prior to the commencement of the study, approval was obtained from the management of each facility, along with the informed, voluntary consent of the participants. Data anonymity and the right to withdraw from participation at any time without consequences were ensured.

### 2.6 Statistical analysis

Statistical analysis was performed using IBM SPSS Statistics software. Quantitative variables were characterized using means, standard deviations, medians, and minimum and maximum values, while categorical variables were presented as frequencies and percentages. The Mann-Whitney U test was used to compare quantitative variables between the resident groups in Poland and Slovakia, and differences in the distribution of categorical variables were assessed using Pearson's chi-square test. Effect size for categorical variables was determined using the Phi coefficient or Cramér's V.

The relationships between the scores of the ADL, IADL, SWLS, and GDS-15 scales and selected aspects of care quality, autonomy, pain, sleep quality, and length of stay in the facility were assessed using Spearman's rank correlation coefficient (ρ). Correlation analyses were conducted separately for the residents of the Polish and Slovakian facilities. All statistical tests were two-tailed, and the level of statistical significance was set at *p* < 0.05.

Analyses were conducted based on the available, complete data for the respective scales. Differences in sample sizes resulted from incomplete responses or the inability to assess selected activities of daily living. Observations with incomplete results for a given scale were excluded only from the analyses involving that scale, without the imputation of missing data. The ADL analysis included 289 individuals (127 from Poland and 162 from Slovakia), and the IADL analysis included 287 individuals (126 and 161, respectively), whereas for the SWLS and GDS-15, the full sample of 322 participants was analyzed.

To account for available potential confounding factors, additional multivariable analyses were performed using a General Linear Model [GLM, Univariate Analysis of Variance (UNIANOVA) procedure]. Separate models were developed for life satisfaction (SWLS) and depressive symptom severity (GDS-15). Each model incorporated the study group (residents of facilities in Poland and Slovakia), sex, length of stay in the facility, and the circumstances surrounding the decision to enter the facility. All explanatory variables were treated as categorical factors. The models included main effects and interactions between factors, up to and including the four-way interaction. The statistical significance of individual effects was assessed using F-tests with Type III sums of squares. Due to the presence of interactions, the interpretation of the main effects required caution. These analyses were conducted for all 322 participants.

Given the exploratory nature of the study and the broad scope of analyzed relationships, a formal correction of the statistical significance level for multiple hypothesis testing was not applied. The presented *p*-values are nominal, and the obtained results were interpreted with caution, taking into account the increased risk of a Type I error. The analyses were treated as a basis for identifying potential relationships that require confirmation in further research.

## 3 Results

### 3.1 Characteristics of the study group

The study included 322 residents of long-term care facilities aged 65 and older, including 127 individuals (39.4%) from Poland and 195 individuals (60.6%) from Slovakia. Characteristics of the study group included sex, length of stay in the current facility, decision-making process regarding the stay, and regular visits from loved ones. In the total study group, 204 participants were women (63.4%) and 118 were men (36.6%). In Poland, women accounted for 78.7% of respondents, whereas in Slovakia they accounted for 53.3%. Sex distribution differed significantly between groups (*p* < 0.001; Phi = 0.258). Length of stay in the facility also differed significantly between countries (*p* < 0.001; Cramér's *V* = 0.235). In Poland, the largest proportion of respondents had stayed in the facility for 6–12 months (33.9%), whereas in Slovakia, individuals staying for longer than 3 years predominated (41.0%).

The decision-making process regarding the stay in the facility differed significantly between groups (*p* = 0.009; Cramér's *V* = 0.171). In Poland, a joint decision made with family was indicated most frequently (59.1%), whereas in Slovakia this response was chosen by 42.1% of respondents. An independent decision to stay was declared more frequently by residents of Slovak facilities (30.3%) than Polish ones (18.9%). Regular visits by loved ones were declared by the majority of residents in both Poland (71.7%) and Slovakia (76.4%). The difference between countries was not statistically significant (*p* = 0.338) (Table 1).

**Table 1:** Characteristics of the study group by country.

Variable	Category	Poland (*n* = 127) *n* (%)	Slovakia (*n* = 195) *n* (%)	*p*	Effect size
Gender	Female	100 (78.7)	104 (53.3)	< 0.001	Phi = 0.258
	Male	27 (21.3)	91 (46.7)		
Length of stay in the facility	2–5 months	16 (12.6)	19 (9.7)	< 0.001	*V* = 0.235
	6–12 months	43 (33.9)	30 (15.4)		
	1–3 years	34 (26.8)	66 (33.8)		
	>3 years	34 (26.8)	80 (41.0)		
Decision regarding stay	Own	24 (18.9)	59 (30.3)	0.009	*V* = 0.171
	Jointly with family	75 (59.1)	82 (42.1)		
	Family or institution	28 (22.0)	54 (27.7)		
Regular visits by loved ones	Yes	91 (71.7)	149 (76.4)	0.338	Phi = 0.053
	No	36 (28.3)	46 (23.6)		

### 3.2 Results of the authors' survey questionnaire

The analysis of the custom section of the questionnaire included the evaluation of living conditions, care organization and staff work, autonomy and co-decision making, social contact and activation, as well as selected aspects of functioning and overall evaluation of care. Individual elements were rated on a scale from 1 to 5, where a higher score indicated a more positive rating of a given aspect, with the exception of the frequency of pain occurrence, where a higher value indicated more frequent pain.

Residents of facilities in Slovakia rated most of the analyzed aspects of living conditions and quality of care higher than residents of facilities in Poland. Significantly higher scores were noted regarding housing conditions (4.25 vs. 3.93; *p* < 0.001), ensuring privacy (4.21 vs. 3.80; *p* < 0.001), and the sense of security (4.55 vs. 4.09; *p* < 0.001). Differences also pertained to the organization of care and contact with staff. Residents of Slovak facilities rated staff availability higher (4.41 vs. 4.09; *p* = 0.001), staff response speed to resident needs (4.34 vs. 3.99; *p* < 0.001), respect and kindness from staff (4.69 vs. 4.31; *p* < 0.001), and clarity of information regarding care and treatment (4.40 vs. 4.10; *p* = 0.001).

The largest differences between the studied groups were observed in the area of autonomy and co-decision making. Residents of facilities in Slovakia declared a greater influence on organizing their own day (4.19 vs. 3.25; *p* < 0.001) and greater participation in making decisions regarding their own care (4.17 vs. 3.37; *p* < 0.001). A similar direction of differences was found in the area of social contact and activation. In the Slovak group, the possibility of regular contact with family and loved ones was rated higher (4.72 vs. 4.09; *p* < 0.001), as was the availability of suitable activities and leisure options (4.55 vs. 3.82; *p* < 0.001), and willingness to participate in proposed activities (4.14 vs. 3.78; *p* < 0.001).

Sleep quality was also rated higher by residents of Slovak facilities (3.92 vs. 3.61; *p* = 0.005). However, no significant difference was found in the frequency of pain occurrence (3.17 vs. 3.20; *p* = 0.853).

The overall rating of care received was significantly higher in the Slovak group (4.44 vs. 3.95; *p* < 0.001). Residents of facilities in Slovakia also demonstrated a greater willingness to recommend their facility to another person in a similar situation (4.49 vs. 4.07; *p* < 0.001).

### 3.3 Results of the standardized questionnaires

As part of the assessment using standardized tools, functional capacity in basic and instrumental activities of daily living (ADL and IADL), life satisfaction (SWLS), and the severity of depressive symptoms (GDS-15) were analyzed. The results were compared between residents of long-term care facilities in Poland and Slovakia.

The mean ADL score in the Polish group was 3.87 points, whereas in the Slovakian group, it was 4.09 points. Despite a slightly higher mean ADL value among the residents of the Slovakian facilities, the difference between the study groups was not statistically significant (*p* = 0.216). Therefore, the obtained results did not provide sufficient evidence to establish differences in functional capacity regarding basic activities of daily living between the residents of the studied facilities in Poland and Slovakia.

Regarding more complex activities of daily living (IADL), residents of the Slovakian facilities obtained a higher mean score than residents of the Polish facilities (3.98 vs. 3.31 points). This difference was statistically significant (*p* = 0.013), indicating a greater level of independence in instrumental activities of daily living within the Slovakian group.

Among the residents of the participating facilities, differences were observed in the levels of life satisfaction and the severity of depressive symptoms. The mean SWLS score was 19.98 points in the group of residents from the three Polish facilities and 23.33 points in the group of residents from the three Slovakian facilities (*p* < 0.001). For the GDS-15, the mean scores were 7.30 and 5.50 points, respectively (*p* < 0.001). The observed differences pertain specifically to the analyzed sample of residents and cannot be interpreted as evidence of analogous differences across the entire populations of long-term care residents in Poland and Slovakia. The GDS-15 results were analyzed as a quantitative variable, without applying cut-off points to categorize symptom severity (Table 2).

**Table 2:** Comparison of functional status, life satisfaction, and depressive symptom severity among residents of the studied long-term care facilities in Poland and Slovakia.

Parameter	ADL (0–6)	IADL (0–8)	SWLS (5–35)	GDS-15 (0–15)
Poland (PL)				
Sample size (*n*)	127	126	127	127
Mean ± SD	3.87 ± 1.94	3.31 ± 2.53	19.98 ± 6.01	7.30 ± 3.66
Median	4.00	3.00	20.00	7.00
Mean rank	138.31	130.35	133.12	188.50
Min.–Max.	0–6	0–8	5–35	0–15
Slovakia (SK)				
Sample size (*n*)	162	161	195	195
Mean ± SD	4.09 ± 2.09	3.98 ± 2.49	23.33 ± 7.16	5.50 ± 3.80
Median	5.00	3.00	24.00	5.00
Mean rank	150.24	154.69	179.98	143.92
Min.–Max.	0–6	0–8	5–35	0–15
Total				
Sample size (*n*)	289	287	322	322
Mean ± SD	4.00 ± 2.03	3.69 ± 2.53	22.01 ± 6.92	6.21 ± 3.84
Median	5.00	3.00	22.00	6.00
Min.–Max.	0–6	0–8	5–35	0–15
Mann–Whitney U	9437.50	8422.50	8778.50	8953.50
*P*	0.216	0.013	< 0.001	< 0.001
*p* (Monte Carlo)	0.213	0.011	< 0.001	< 0.001

ADL, Activities of daily living—scale assessing basic activities of daily living; IADL, Instrumental activities of daily living—scale assessing instrumental activities of daily living; SWLS, Satisfaction With Life Scale—scale assessing satisfaction with life; GDS-15, 15-item Geriatric Depression Scale−15-item scale assessing depressive symptoms in older adults. Differences in sample sizes result from incomplete data regarding ADL and IADL. Missing data were not imputed. Between-group comparisons were performed using the Mann–Whitney U-test. The presented p-values were not corrected for multiple hypothesis testing. Values of p < 0.05 are highlighted.

### 3.4 Multivariable analysis

In the multivariable analysis incorporating the study group (residents of facilities in Poland and Slovakia), sex, length of stay in the facility, and the circumstances of the admission decision, along with interactions between these variables, the main effect test for the group in the life satisfaction (SWLS) model reached statistical significance (*p* = 0.002). For the severity of depressive symptoms (GDS-15), the main effect test for the group did not reach statistical significance (*p* = 0.078).

In the SWLS model, significant interactions were found between the group and sex (*p* = 0.013), group and length of stay (*p* = 0.045), and among group, sex, and circumstances of the admission decision (*p* = 0.013). In the GDS-15 model, a significant interaction was observed among the group, length of stay, and circumstances of the admission decision (*p* = 0.033), as well as a four-way interaction involving all analyzed factors (*p* = 0.038).

The presence of significant interactions indicates that the relationships between the study group and the SWLS and GDS-15 scores may vary depending on the other incorporated factors. Therefore, the significance of the main effects for the group should not be interpreted as confirmation of uniform differences between the residents of the Polish and Slovakian facilities across all analyzed subgroups. Detailed results of the multivariable analyses are presented in Table 3.

**Table 3:** Multivariable analysis of life satisfaction (SWLS) and depressive symptom severity (GDS-15) among residents of the six studied facilities.

Effect	*df*	SWLS: *F*	SWLS: *p*	GDS-15: *F*	GDS-15: *p*
Overall model	45	2.289	< 0.001	2.084	< 0.001
Study group (PL/SK)	1	9.902	0.002	3.139	0.078
Gender	1	0.574	0.449	1.322	0.251
Length of stay	3	0.397	0.756	0.894	0.445
Circumstances of admission decision	2	1.135	0.323	0.607	0.546
Group × gender	1	6.313	0.013	1.270	0.261
Group × length of stay	3	2.718	0.045	1.745	0.158
Group × circumstances of decision	2	0.831	0.437	0.240	0.786
Gender × length of stay	3	1.545	0.203	2.425	0.066
Gender × circumstances of decision	2	0.865	0.422	2.021	0.135
Length of stay × circumstances of decision	6	0.871	0.517	1.674	0.127
Group × gender × length of stay	3	0.638	0.591	0.474	0.701
Group × gender × circumstances of admission decision	2	4.434	0.013	0.409	0.665
Group × length of stay × circumstances of decision	6	0.397	0.881	2.329	0.033
Gender × length of stay × circumstances of decision	6	1.613	0.143	2.108	0.053
Group × gender × length of stay × circumstances of decision	4	1.113	0.350	2.570	0.038

PL, facilities in Poland; SK, facilities in Slovakia; SWLS, Satisfaction with Life Scale; GDS-15, 15-item Geriatric Depression Scale; df, degrees of freedom; F, F-statistic; R^2^, coefficient of determination. Presented are the results of tests of between-subjects effects in the full General Linear Models (GLM/UNIANOVA), incorporating the study group, sex, length of stay in the facility, circumstances of the admission decision, and interactions between these factors. Type III sums of squares were used. In both models, N = 322; error df = 276. The coefficients of determination refer to the entire models, rather than individual effects: SWLS: R^2^ = 0.272; adjusted R^2^ = 0.153; GDS-15: R^2^ = 0.254; adjusted R^2^ = 0.132. Due to the presence of interactions, the main effects of the group should not be interpreted as confirmation of uniform differences between the study groups across all subgroups.

### 3.5 Correlations

The conducted correlation analyses were exploratory in nature and aimed to identify potential relationships between the analyzed variables. Associations were observed among functional capacity, life satisfaction, and the severity of depressive symptoms in both study groups. Due to the absence of a correction for multiple hypothesis testing, the presented *p*-values are nominal; therefore, the results should be interpreted primarily by considering the direction and strength of the correlations.

In the Polish group, a strong positive correlation was found between ADL and IADL (ρ = 0.691; *p* < 0.001), indicating that greater independence in basic activities of daily living was accompanied by greater capacity in performing complex activities. Both ADL (ρ = 0.190; *p* = 0.032) and IADL (ρ = 0.272; *p* = 0.002) were positively associated with life satisfaction, although the strength of these relationships was weak. A higher level of functional capacity was also associated with lower severity of depressive symptoms—for ADL ρ = −0.214 (*p* = 0.016), and for IADL ρ = – 0.218 (*p* = 0.014). The strongest relationship regarding psychological wellbeing was recorded between SWLS and GDS-15 (ρ = −0.528; *p* < 0.001), meaning that higher life satisfaction was accompanied by lower severity of depressive symptoms.

In the Slovak group, a positive correlation between ADL and IADL was also demonstrated (ρ = 0.567; *p* < 0.001). Higher ADL scores (ρ = 0.180; *p* = 0.022) and IADL scores (ρ = 0.197; *p* = 0.012) were weakly, but significantly, associated with greater life satisfaction. At the same time, greater functional capacity was associated with lower severity of depressive symptoms. This relationship was more pronounced for ADL (ρ = −0.381; *p* < 0.001) than for IADL (ρ = −0.231; p = 0.003). Similar to the Polish group, the strongest relationship regarding psychological wellbeing was found between SWLS and GDS-15 (ρ = −0.535; *p* < 0.001).

In both studied groups, the direction of the analyzed associations was consistent: greater functional independence was accompanied by higher life satisfaction and lower severity of depressive symptoms. Although numerical differences in correlation coefficients were observed between the Polish and Slovak groups, formal significance testing to compare these coefficients was not performed. Consequently, it cannot be determined whether the differences in the strength of these associations between the groups were statistically significant (Table 4).

**Table 4:** Relationships between functional capacity, life satisfaction, and severity of depressive symptoms in the Polish (*n* = 127) and Slovak (*n* = 195) groups.

Group		Analyzed variable	Correlation coefficient	Assessment of basic activities of daily living (ADL) (0–6)	Assessment of instrumental activities of daily living (IADL) (0–8)	Satisfaction with life (SWLS) (5–35)	Geriatric Depression Scale (GDS-15) (0–15)
Spearman's rho	PL	Assessment of basic activities of daily living (ADL) (0–6)	Correlation coefficient	1.000			
			Significance (two-tailed)				
		Assessment of instrumental activities of daily living (IADL) (0–8)	Correlation coefficient	0.691^**^	1.000		
			Significance (two-tailed)	< 0.001			
		Satisfaction with life (SWLS) (5–35)	Correlation coefficient	0.190^*^	0.272^**^	1.000	
			Significance (two-tailed)	0.032	0.002		
		Geriatric Depression Scale (GDS-15) (0–15)	Correlation coefficient	−0.214^*^	−0.218^*^	−0.528^**^	1.000
			Significance (two-tailed)	0.016	0.014	< 0.001	
	SK	Assessment of basic activities of daily living (ADL) (0–6)	Correlation coefficient	1.000			
			Significance (two-tailed)				
		Assessment of instrumental activities of daily living (IADL) (0–8)	Correlation coefficient	0.567^**^	1.000		
			Significance (two-tailed)	< 0.001			
		Satisfaction with life (SWLS) (5–35)	Correlation coefficient	0.180^*^	0.197^*^	1.000	
			Significance (two-tailed)	0.022	0.012		
		Geriatric Depression Scale (GDS-15) (0–15)	Correlation coefficient	−0.381^**^	−0.231^**^	−0.535^**^	1.000
			Significance (two-tailed)	< 0.001	0.003	< 0.001	

^**^Correlation significant at the 0.01 level (two-tailed) ^*^Correlation significant at the 0.05 level (two-tailed) The presented p-values were not corrected for multiple hypothesis testing. The analyses were exploratory in nature, and the observed relationships require confirmation in future studies.

### 3.6 Relationships between quality of care, autonomy, functional capacity and psychosocial well-being of residents

In the analysis of the relationships between the subjective evaluation of quality of care and autonomy and the psychosocial functioning of residents, different correlation patterns were found in the Polish and Slovak groups. In the Polish group, life satisfaction was positively associated with most of the analyzed aspects of care experience. The highest coefficient values were recorded for ensuring privacy (ρ = 0.431; *p* < 0.001), the possibility of regular contact with family and loved ones (ρ = 0.421; *p* < 0.001), sense of security (ρ = 0.385; *p* < 0.001), housing conditions (ρ = 0.351; *p* < 0.001), and staff response speed to resident needs (ρ = 0.346; *p* < 0.001). Positive relationships with SWLS were also found for clarity of information regarding care and treatment (ρ = 0.336; *p* < 0.001), participation in proposed activities (ρ = 0.324; *p* < 0.001), staff availability (ρ = 0.292; *p* = 0.001), overall evaluation of care (ρ = 0.273; *p* = 0.002), and respect and kindness from staff (ρ = 0.269; *p* = 0.002).

In the Polish group, resident autonomy was also associated with the analyzed outcomes. A greater possibility of influencing the organization of one's own day correlated positively with IADL (ρ = 0.232; p = 0.009) and SWLS (ρ = 0.233; *p* = 0.008), whereas greater participation in decisions regarding one's own care was associated with higher IADL (ρ = 0.250; *p* = 0.005) and SWLS (ρ = 0.259; *p* = 0.003) scores. Regarding depressive symptoms, significant negative relationships were found primarily for regular contact with family and loved ones (ρ = −0.301; *p* = 0.001), participation in proposed activities (ρ = −0.297; *p* = 0.001), availability of suitable activity programs (ρ = −0.205; *p* = 0.021), and sense of security (ρ = −0.191; *p* = 0.031).

In the Slovak group, life satisfaction was also positively associated with numerous aspects of quality of care and autonomy. The highest coefficient value was recorded for the overall evaluation of care (ρ = 0.405; *p* < 0.001), followed by the availability of suitable activity programs (ρ = 0.366; *p* < 0.001), participation in decisions regarding one's own care (ρ = 0.334; *p* < 0.001), respect and kindness from staff (ρ = 0.318; *p* < 0.001), and the possibility of influencing the organization of one's own day (ρ = 0.308; *p* < 0.001). Higher evaluations of these care aspects were thus associated with greater life satisfaction.

In the Slovak group, more consistent negative relationships were also observed between care evaluation and the severity of depressive symptoms. A higher rating of activity program availability was associated with lower GDS-15 scores (ρ = −0.309; *p* < 0.001), as was greater staff availability (ρ = −0.304; *p* < 0.001), respect and kindness from staff (ρ = −0.294; p < 0.001), a higher overall evaluation of care (ρ = −0.264; *p* < 0.001), and greater participation in decisions regarding one's own care (ρ = −0.243; *p* = 0.001). The possibility of influencing the organization of one's own day was also associated with lower severity of depressive symptoms (ρ = −0.180; *p* = 0.012). Notable are the relationships regarding autonomy in the Slovak group. Both the possibility of influencing daily organization and participation in care decisions were positively associated with ADL capacity (ρ = 0.284 and ρ = 0.288, respectively; both *p* < 0.001) and IADL capacity (ρ = 0.261, *p* = 0.001 and ρ = 0.282, *p* < 0.001, respectively). At the same time, both autonomy aspects were linked to higher life satisfaction and lower severity of depressive symptoms.

Overall, the results indicate that in both groups, a more positive evaluation of selected care aspects and greater resident autonomy were primarily associated with higher life satisfaction. Relationships with the severity of depressive symptoms were more consistently observed in the Slovak group, whereas in the Polish group, contact with loved ones and participation in activities played a particularly important role.

### 3.7 Pain and sleep quality

In the Polish group, a higher frequency of pain was significantly associated with lower basic functional capacity (ADL; ρ = −0.199; *p* = 0.025) and instrumental activities of daily living (IADL; ρ = −0.223; *p* = 0.012), lower life satisfaction (SWLS; ρ = −0.273; *p* = 0.002), and greater severity of depressive symptoms (GDS-15; ρ = 0.220; *p* = 0.013). However, no significant relationships were found between sleep quality and ADL, IADL, SWLS, or GDS-15.

In the Slovak group, a higher frequency of pain was significantly associated only with greater severity of depressive symptoms (GDS-15; ρ = 0.191; *p* = 0.007). No significant relationships were demonstrated between pain frequency and ADL, IADL, and SWLS. On the other hand, a higher rating of sleep quality was associated with greater life satisfaction (SWLS; ρ = 0.255; *p* < 0.001) and lower severity of depressive symptoms (GDS-15; ρ = −0.221; *p* = 0.002), with no significant relationships found with ADL and IADL (Table 5).

**Table 5:** Relationships between pain frequency, sleep quality, functional capacity, life satisfaction, and severity of depressive symptoms in the Polish and Slovak groups.

Group		Analyzed variable	Correlation coefficient	Assessment of basic activities of daily living (ADL) (0–6)	Assessment of instrumental activities of daily living (IADL) (0–8)	Satisfaction with life (SWLS) (5–35)	Geriatric depression scale (GDS-15) (0–15)
Spearman's rho	PL	How often do you experience pain?	Correlation coefficient	−0.199^*^	−0.223^*^	−0.273^**^	0.220^*^
			Significance (two-tailed)	0.025	0.012	0.002	0.013
		How do you rate the quality of sleep in the facility?	Correlation coefficient	0.068	0.120	0.149	−0.095
			Significance (two-tailed)	0.447	0.180	0.094	0.288
	SK	How often do you experience pain?	Correlation coefficient	−0.073	0.015	−0.055	0.191^**^
			Significance (two-tailed)	0.358	0.850	0.446	0.007
		How do you rate the quality of sleep in the facility?	Correlation coefficient	−0.038	−0.024	0.255^**^	−0.221^**^
			Significance (two-tailed)	0.633	0.764	0.000	0.002

^**^Correlation significant at the 0.01 level (two-tailed) ^*^Correlation significant at the 0.05 level (two-tailed).

Length of stay in the facility was not significantly associated with functional capacity measured by ADL and IADL, life satisfaction, or the severity of depressive symptoms in either the Polish or Slovak group (all *p* > 0.05).

## 4 Discussion

In the studied sample, differences were observed between residents of the participating long-term care facilities in Poland and Slovakia regarding subjective evaluations of living conditions and care quality, autonomy, life satisfaction, and the severity of depressive symptoms. Given the limited number of facilities and the inability to account for clustering effects within institutions, these findings should not be interpreted as representative differences between the national long-term care systems of the two countries. The observed differences among the residents of the participating facilities should be interpreted in the context of the specific nature of the studied institutions and the characteristics of their residents. The higher ratings of selected aspects of care quality and autonomy, greater life satisfaction, and lower severity of depressive symptoms in the Slovakian group do not provide a basis for concluding that analogous differences exist across the entire long-term care systems of both countries. These findings may partially reflect the distinct organizational, environmental, and staffing conditions of the individual facilities, as well as differences in participant characteristics. Because facility identifiers were not retained in the analytical dataset, it was not possible to determine the extent to which the observed differences were associated with the residents' placement in specific institutions. Therefore, the obtained results should be treated as observations pertaining solely to the six participating facilities, serving as a starting point for further comparative research.

Residents of Slovak facilities rated most of the analyzed aspects of the care environment higher, including living conditions, privacy, security, availability and actions of staff, the ability to maintain social contacts, and the availability of activities. Particularly clear differences concerned autonomy—the ability to influence the organization of one's own day and participation in decisions regarding one's own care. At the same time, residents of Slovak facilities were characterized by higher life satisfaction and lower severity of depressive symptoms. These results indicate the need to consider the wellbeing of LTC residents not only in the context of their health status and functional capacity, but also their daily experience of care and the ability to maintain control over their own lives.

Such an approach aligns with the contemporary understanding of the quality of long-term care. Literature reviews indicate that the quality of life of residents remains linked to both individual factors and potentially modifiable features of the facility's environment (3, 4). In a systematic review by Kawashima et al., it was shown that social support, the ability to participate in activities, and the characteristics of the facility environment can be significant for maintaining or improving the quality of life and wellbeing of older adults, alongside individual factors such as independence in activities of daily living (4). The relationships obtained in this study between life satisfaction and the evaluation of living conditions, security, privacy, staff availability, and activation thus fit into a broader model of LTC quality, in which the institutional environment constitutes an integral element of resident wellbeing.

Resident autonomy is of particular importance in the results obtained. In the Slovak group, both the ability to influence the organization of one's own day and participation in decisions regarding one's own care were rated significantly higher than in the Polish group. In both groups, greater autonomy was associated with higher life satisfaction, whereas in the Slovak group, both analyzed dimensions were also associated with lower severity of depressive symptoms and greater functional capacity. These results are consistent with the concept of person-centered care, according to which autonomy should not be identified solely with physical independence. Even a resident requiring assistance in performing daily tasks can retain the ability to make choices, express preferences, and participate in decisions regarding their own care (5–7). Van Loon et al. emphasize that supporting resident autonomy requires not only appropriate staff attitudes, but also organizational solutions that enable real participation of the older adult in shaping daily life (5). Conversely, observations by Hoek et al. indicate that opportunities to support autonomy during daily staff-resident interactions are not always fully utilized (6). The importance of this area is also confirmed by a recent review of person-centered care models, in which autonomy and dignity, individualization of care, social engagement, a home-like environment, and family involvement were identified as key components of person-centered care (7). Our results find additional support in a study by Nigg et al. conducted among 154 residents of 16 long-term care facilities. The authors demonstrated relationships between perceived person-centered care, a sense of control, and life satisfaction, while also pointing out the importance of resident participation in daily decisions (32). From the residents' perspective, autonomy and the ability to control one's own life are also elements that build a sense of “homeliness” in the facility. In a qualitative meta-analysis by Gan et al., covering 19 studies and the perspective of 1,368 residents, four fundamental dimensions of a favorable care home environment were identified: psychological, social, spatial, and organizational. Key elements included autonomy and control, relationships with staff and family, private space, social activities, and care based on respect (33). These findings closely align with the areas that, in our study, remained associated with residents' life satisfaction.

Significant results of the study also included differences in life satisfaction and depressive symptoms. Residents of Slovak facilities achieved higher SWLS scores, whereas in the Polish group, greater severity of depressive symptoms measured by the GDS-15 was observed. These results should be considered within the context of the multifactorial determinants of LTC residents' psychological wellbeing. Previous studies in the population of older adults receiving institutional care indicated associations between quality of life and degree of independence (8), as well as life satisfaction with individual psychological resources and health behaviors (9). In turn, studies on depressive symptoms indicate that they can persist for a long period after admission to a facility (10), and in the Polish LTC resident population, their occurrence was associated with factors such as limited contact with family, multimorbidity, polypharmacy, and lower functional independence (11).

The importance of functional capacity is also confirmed by the results of this study. In both the Polish and Slovak groups, higher ADL and IADL scores were associated with higher life satisfaction and lower severity of depressive symptoms. At the same time, a clear negative correlation was found between SWLS and GDS-15 in both groups. These results are consistent with observations by Swakowska et al., who, in a study of care home residents in Poland and Germany, demonstrated a relationship between quality of life and the subjects' degree of independence (8). Likewise, a recent review of longitudinal studies identifies independence in activities of daily living as one of the individual factors associated with changes in quality of life and wellbeing among facility residents (4). However, it should be stressed that autonomy and functional capacity represent distinct constructs. Dependency on help with ADL or IADL should not automatically mean depriving a resident of the ability to make choices and co-decide about daily life.

In the context of evaluating functional status in older adults, the Barthel Index is also noteworthy; like Katz's ADL scale, it measures independence in performing basic activities of daily living. The importance of evaluating functional outcomes and patient autonomy in intermediate care settings has also been highlighted by (34). However, it should be noted that functional independence and decisional autonomy represent distinct, albeit interrelated, dimensions of human functioning. While the Barthel Index primarily assesses the degree of dependency on others for daily activities, it does not directly measure an individual's capacity to participate in decision-making regarding their own life and care (34).

An important element of the obtained results is also the social and relational aspects of staying in a facility. In the Polish group, the possibility of regular contact with family and loved ones was among the aspects most strongly associated with higher life satisfaction, while also being associated with lower severity of depressive symptoms. Favorable relationships were also found for participation in proposed activities. In the Slovak group, significant factors included the availability of activities, staff availability, and respect and kindness in staff relations. The importance of these factors is consistent with findings by Kuven et al., indicating that nurse-resident interactions are an important element of quality of life, whereas loneliness can adversely affect the wellbeing of individuals residing in facilities (16). The relational dimension of care is particularly important in the case of residents with depressive symptoms. Schweighart et al. demonstrated that residents' ability to communicate needs depends on staff availability, patience, willingness to listen, and treating the resident as a partner (17). Furthermore, qualitative studies on the experiences of older adults show that care perceived as lacking respect can be associated with feelings of insignificance, sadness, and suffering (18). In this context, the observed associations of life satisfaction and depressive symptoms with staff availability, respect, kindness, and quality of communication in our study should be treated as an argument for incorporating relational aspects of care into LTC quality assessments.

Differences regarding social contacts and activation also deserve attention. In the Polish group, regular contact with loved ones was associated with both higher life satisfaction and lower severity of depressive symptoms, while participation in activities showed a similar direction of association. These results correspond with current models of person-centered care, in which social engagement and family involvement are treated as integral elements of care responding to the resident's individual needs (7). The synthesis by Gan et al. also indicates that family relationships, social interactions, and the availability of meaningful activities belong to the features of an environment that promotes a sense of security and “feeling at home” in the facility (33). In practice, this means that resident activation should not be treated merely as an additional form of time management, but as a potentially key element of their daily wellbeing.

Results concerning pain and sleep quality further point to the need for a multidimensional evaluation of LTC residents. In the Polish group, more frequent pain experience was associated with lower basic functional capacity in ADL and IADL, lower life satisfaction, and greater severity of depressive symptoms. In the Slovak group, pain was associated with greater severity of depressive symptoms, whereas better sleep quality was associated with higher life satisfaction and lower severity of depressive symptoms. Similar interrelationships between pain, sleep, depressive symptoms, and care dependency were described by Puto et al. among older adults in a long-term care facility (35). In their study, deterioration of sleep quality was associated with greater pain severity, greater ADL dependency, depressive symptoms, and greater care dependency. The connection between these areas is also confirmed by other studies of nursing home populations. Hou demonstrated the co-occurrence and mutual relationships of depressive symptoms, poorer sleep quality, pain, and frailty syndrome in nursing home residents (36). In turn, Collins et al., analyzing data from 1,589 care home residents, found a high prevalence of pain and anxiety or depression, with the most favorable quality of life observed in residents without pain, whereas poorer quality of life co-occurred with greater pain severity and a higher burden of symptomatic treatment (37). These findings reinforce the importance of regular pain and sleep assessment as components of comprehensive geriatric care, especially when symptoms of low mood or functional limitations co-occur.

It is worth noting that length of stay in the facility was not significantly associated with functional capacity, life satisfaction, or the severity of depressive symptoms in either analyzed group in our study. This result may indicate that the length of time spent in an institution does not simply reflect resident wellbeing. Current functional capacity, health status, social relationships, and the experience of daily care may be more critical. The complexity of the adaptation process to living in a facility and the potential for quality of life to change over time are also confirmed by longitudinal studies (4, 10). However, due to the cross-sectional nature of this study, it is not possible to determine the direction of these relationships.

A direct comparison of the results obtained in Poland and Slovakia also requires considering the broader organizational and social context. Long-term care systems differ in organization, funding, and the integration of health and social services. Literature concerning Slovakia has drawn attention to the transformation process of social services and issues related to the integration and coordination of long-term care (19, 20). At the same time, a previous comparative study of community-dwelling seniors in Poland and Slovakia also demonstrated cross-country differences in quality of life and its individual dimensions (21). The present study extends this perspective to the population of 24-h LTC facility residents, showing that differences concern not only wellbeing, but also the subjective experience of care quality and autonomy. However, differences between the groups should not be interpreted as direct evidence of a higher quality of the entire long-term care system in one of the countries. The study included residents of six facilities, and the groups differed in terms of gender structure, length of stay, and the decision-making process regarding institutionalization. Furthermore, the cross-sectional design of the study does not allow establishing causality for the observed relationships. Thus, it cannot be asserted that greater autonomy or a more positive evaluation of care lead to higher life satisfaction or lower severity of depressive symptoms. The reverse relationship is also possible, wherein individuals with better psychological wellbeing rate the facility environment and staff relationships more favorably.

Despite these limitations, the study results support a multidimensional approach to long-term care quality. Functional capacity remains an important element of resident wellbeing, but it does not fully encompass their needs. Autonomy, co-decision making, privacy, sense of security, relationships with staff and family, and access to meaningful activities represent elements of the resident's daily experience that can be modified at the organizational level (4, 5, 7, 32, 33). From a geriatric care perspective, the results advocate for the further development of person-centered care models, in which the evaluation of service quality is supplemented by assessing the extent to which the facility environment allows residents to maintain personhood, social ties, and the greatest possible control over their daily lives.

Due to the cross-sectional and observational design of the study, the observed associations between the subjective evaluation of care, residents' autonomy, and their psychosocial wellbeing do not allow for determining the directionality of these relationships or inferring causality. Therefore, it cannot be concluded that greater autonomy or a more positive evaluation of care directly contributes to increased life satisfaction or a reduction in the severity of depressive symptoms. However, the obtained results underscore the need for further investigation into these associations and may provide a basis for formulating hypotheses regarding the potential significance of person-centered care for the wellbeing of residents in long-term care facilities. Verifying these hypotheses will require longitudinal and interventional studies that account for potential confounding factors.

## 5 Conclusions

1. Among the residents of the six long-term care facilities participating in the study, differences were observed in the subjective evaluation of selected aspects of care quality and autonomy, life satisfaction, and the severity of depressive symptoms. Residents of the three studied facilities in Slovakia more frequently reported positive experiences related to care and shared decision-making, and they also obtained higher life satisfaction scores and lower scores for depressive symptom severity than residents of the three studied facilities in Poland.
2. In both analyzed groups, associations were observed between functional capacity, selected aspects of the subjective evaluation of care and autonomy, and the psychosocial wellbeing of the residents. The obtained associations were unadjusted and do not allow for determining their directionality or the independent impact of individual factors.
3. The results highlight the importance of considering not only the health and functional needs of residents in the assessment of long-term care, but also their autonomy, opportunities for shared decision-making, social relationships, and participation in the daily life of the facility. These observations provide a rationale for further research into the significance of person-centered care for resident wellbeing.
4. Due to the purposive sampling of the six facilities, the lack of institution identifiers to account for clustered observations, and the exploratory nature of the analyses, the obtained results must be interpreted exclusively in relation to the residents of the participating facilities. They do not provide a basis for drawing representative conclusions regarding differences between the national long-term care systems in Poland and Slovakia. Confirming the observed associations requires further studies involving a larger number of facilities, taking into account their characteristics, the clustered structure of the data, and potential confounding factors.

### 5.1 Limitations of the study

This study has several methodological limitations that should be considered when interpreting the results. First and foremost, its cross-sectional design precludes determining the direction of the observed associations and drawing causal inferences. Furthermore, the study was conducted in six non-randomly selected long-term care facilities (three each in Poland and Slovakia), which limits the generalizability of the findings to the national long-term care systems. An additional limitation was the unequal participation rate in both countries, which may increase the risk of selection bias.

A significant limitation was the presence of differences between the study groups regarding sex distribution, length of stay, and the circumstances surrounding the decision to reside in the facility. While these variables were incorporated into the additional multivariable analyses, the complex structure of the models, which included numerous interactions, limits the straightforward interpretation of the main effects. Moreover, detailed data regarding the age of the participants were not collected, precluding the inclusion of this variable in the analyses. Therefore, the impact of other unmeasured confounding factors on the obtained results cannot be ruled out.

Consequently, the presented comparisons apply exclusively to the residents of the six participating facilities and do not allow for the formulation of representative conclusions regarding differences in care quality, autonomy, or resident wellbeing between the national long-term care systems in Poland and Slovakia. Additionally, the small number of facilities and potentially small subgroup sizes limited the ability to conduct more detailed analyses at the individual institution level.

Another limitation was the lack of facility identifiers in the analytical dataset, which precluded evaluating the clustering effect of observations and applying methods that account for the non-independence of outcomes among residents of the same institution. Thus, the influence of organizational, staffing, and environmental differences among the facilities on the obtained results cannot be excluded. The failure to account for the clustered data structure could also have affected the estimation of statistical uncertainty and the significance level of the observed associations. Furthermore, with a small number of facilities, it is impossible to definitively separate country-level differences from institution-specific conditions.

Another limitation was the use of a custom-developed questionnaire assessing selected aspects of care quality and resident autonomy. Despite undergoing linguistic and cultural adaptation involving forward and backward translation, the tool was not subjected to formal psychometric validation, expert evaluation of content validity, or pilot testing. Therefore, the obtained results should be treated as subjective evaluations of selected care aspects rather than measurements based on a validated scale. The self-report nature of a portion of the data also carried a risk of being influenced by current mood, individual expectations, and social desirability bias.

Interpreting the IADL scale results requires considering the specific nature of institutional care, where some activities of daily living are routinely performed by the staff. Consequently, the non-performance of these activities by residents does not necessarily result solely from functional limitations but may also be a consequence of care organization.

Finally, a significant limitation of the study is the conduction of numerous statistical comparisons and correlation analyses without applying a correction for multiple hypothesis testing, which increases the risk of obtaining false-positive results (Type I error). This strategy stemmed from the exploratory nature of the study, aimed at identifying potential patterns and relationships rather than definitively verifying pre-specified hypotheses. Forgoing the correction allowed for maintaining greater analytical sensitivity to potential associations; however, it limits the certainty of result interpretation, particularly for weak correlations and *p*-values close to the adopted significance level. To ensure transparency, the analysis results are presented alongside the correlation coefficient values and statistical significance levels. Moreover, formal tests comparing the strength of correlations between the national groups were not conducted. Therefore, the obtained results should be treated as preliminary observations requiring corroboration in future confirmatory studies that account for appropriate control of multiple testing, potential confounding factors, and the clustered structure of the data.

### 5.2 Implications for practice

The obtained results point to the need for incorporating not only residents' functional capacity and health status into long-term care, but also their autonomy, opportunities for co-decision making, and subjective experience of daily care. In practice, this supports the rationale for encouraging residents to make choices regarding their daily schedule, the manner of care delivery, and participation in available activities, even for individuals requiring assistance with ADLs and IADLs.

Strengthening the resident's relationships with staff and loved ones should also constitute an essential element of care quality. Staff availability, respect, kind communication, maintaining family ties, and the opportunity to engage in meaningful activities can represent important target areas in organizing person-centered care.

Furthermore, the results highlight the justification for comprehensive and regular evaluation of resident wellbeing, encompassing depressive symptoms, pain, and sleep quality alongside functional capacity. Individuals with limited independence, severe pain, sleep disturbances, or restricted social contacts may require special attention. Incorporating the resident's perspective into care quality monitoring can facilitate the identification of areas requiring individualization and support the development of person-centered care in LTC facilities.

## Data Availability

The raw data supporting the conclusions of this article will be made available by the authors, without undue reservation.
